# Squamous Cell Carcinoma Arising in a Mature Cystic Teratoma of the Ovary: A Case Report

**DOI:** 10.7759/cureus.117733

**Published:** 2026-10-10

**Authors:** Shivani Sharma, Meeta Gupta, Tania Verma, Natasha Gupta, Vanessa Ravel

**Affiliations:** 1 Obstetrics and Gynaecology, All India Institute of Medical Sciences, Vijaypur, Jammu, IND; 2 Public Health, Private Practice, Chennai, IND

**Keywords:** germ cell tumor, malignant transformation, mature cystic teratoma (mct), ovarian dermoid cyst, squamous cell carcinoma (scc)

## Abstract

Mature cystic teratoma (MCT) is a common benign ovarian germ cell tumor, although malignant transformation occurs rarely in 1-2% of cases, most frequently into squamous cell carcinoma (SCC). Bilateral MCTs with malignant transformation occurring in only one ovary are uncommon. A 46-year-old perimenopausal woman presented with incomplete bladder emptying and lower abdominal pain. Clinical evaluation revealed several features associated with malignant transformation, including age >40 years, a large mass (12.2 cm), bilateral MCTs, and markedly elevated cancer antigen 125 (CA-125) at 185.9 U/mL. The patient underwent total abdominal hysterectomy with bilateral salpingo-oophorectomy (TAH-BSO), with histopathology confirming malignant transformation into moderately differentiated SCC within the left ovarian MCT capsule, with intact tumor protein 63 (p63) and p40 nuclear positivity supporting the squamous phenotype, alongside a benign right ovarian MCT. Postoperative positron emission tomography-computed tomography (PET-CT) showed no evidence of metastatic disease, and serum CA-125 decreased to 35.0 U/mL. She subsequently received adjuvant carboplatin and paclitaxel chemotherapy and remained without evidence of disease at 6-month follow-up. This case highlights the importance of considering malignant transformation in perimenopausal women with large, bilateral MCTs and elevated tumor markers.

## Introduction

Mature cystic teratoma (MCT), also known as a dermoid cyst, is the most common benign ovarian tumor, comprising approximately 10-20% of all ovarian neoplasms, particularly in women of reproductive age. The histological structure of MCT consists of at least two mature germ layers (ectoderm, mesoderm, and endoderm). Ectodermal and mesodermal germ cells are the most frequently occurring germ cells in the wall of the tumour; hence, the mature structures of the skin, hair (ectodermal), fat, and muscle (mesodermal) constitute the composition of MCT. These tumors are typically unilateral; however, bilateral involvement is observed in approximately 10-12% of cases [1-4]. Malignant transformation of MCTs is a rare occurrence, reported in approximately 1-2% of cases, with squamous cell carcinoma (SCC) being the most frequently encountered histological type [1,5-6].

Preoperative identification of malignant transformation remains challenging because clinical symptoms and imaging findings may mimic those of benign teratomas. Clinical features associated with malignant transformation include age >40 years, tumor diameter >10 cm, bilateral MCTs, and elevated tumor markers. Nonetheless, diagnosis prior to surgery is difficult since the clinical and radiographic characteristics of transformation to malignancy can be similar to those of benign MCT [5,7].

The presence of bilateral MCTs with malignant transformation in only one ovary is rare but carries diagnostic and treatment dilemmas. Here we present a case of bilateral MCTs in a 46-year-old female patient, with malignant transformation to moderately differentiated SCC in the left ovarian teratoma. It demonstrates the necessity of meticulous investigation of MCTs, especially in females older than 40 years, where tumors are large or have increased tumor markers.

## Case presentation

A 46-year-old lady at the perimenopause stage (Para 2, Live 2, Abortion 2), with a history of vaginal delivery, was referred to the gynecology outpatient clinic with symptoms of incomplete bladder emptying lasting three months and lower abdominal pain lasting two months. She had regular menstrual cycles and a history of hypothyroidism. Her vital signs were stable, and no peripheral lymphadenopathy was noted. Breast examination was unremarkable.

Abdominal examination revealed a soft, mobile, non-tender pelvic mass with smooth borders extending to approximately 16 weeks' gestation size. Speculum examination showed a normal-appearing cervix displaced anteriorly. On bimanual vaginal examination, uterine size could not be delineated due to full bilateral fornices and a soft-to-firm, mobile, non-tender mass occupying the posterior fornix and pouch of Douglas. Digital rectal examination confirmed intact rectal mucosa.

The findings on pelvic ultrasonography were a uterine size of 10.4 × 4.2 × 5.8 cm. There was an irregularly shaped heteroechoic cystic mass of size 5.1 × 4.5 × 4.8 cm with hyperechoic material without posterior acoustic shadowing, found in the right adnexa. Similarly, there was another well-defined heteroechoic cystic mass of size 10.8 × 10.4 × 11.4 cm in the left adnexa. Both masses exhibited internal "dot-and-dash" hyperechoic patterns characteristic of MCTs (Figure 1).

**Figure 1:**
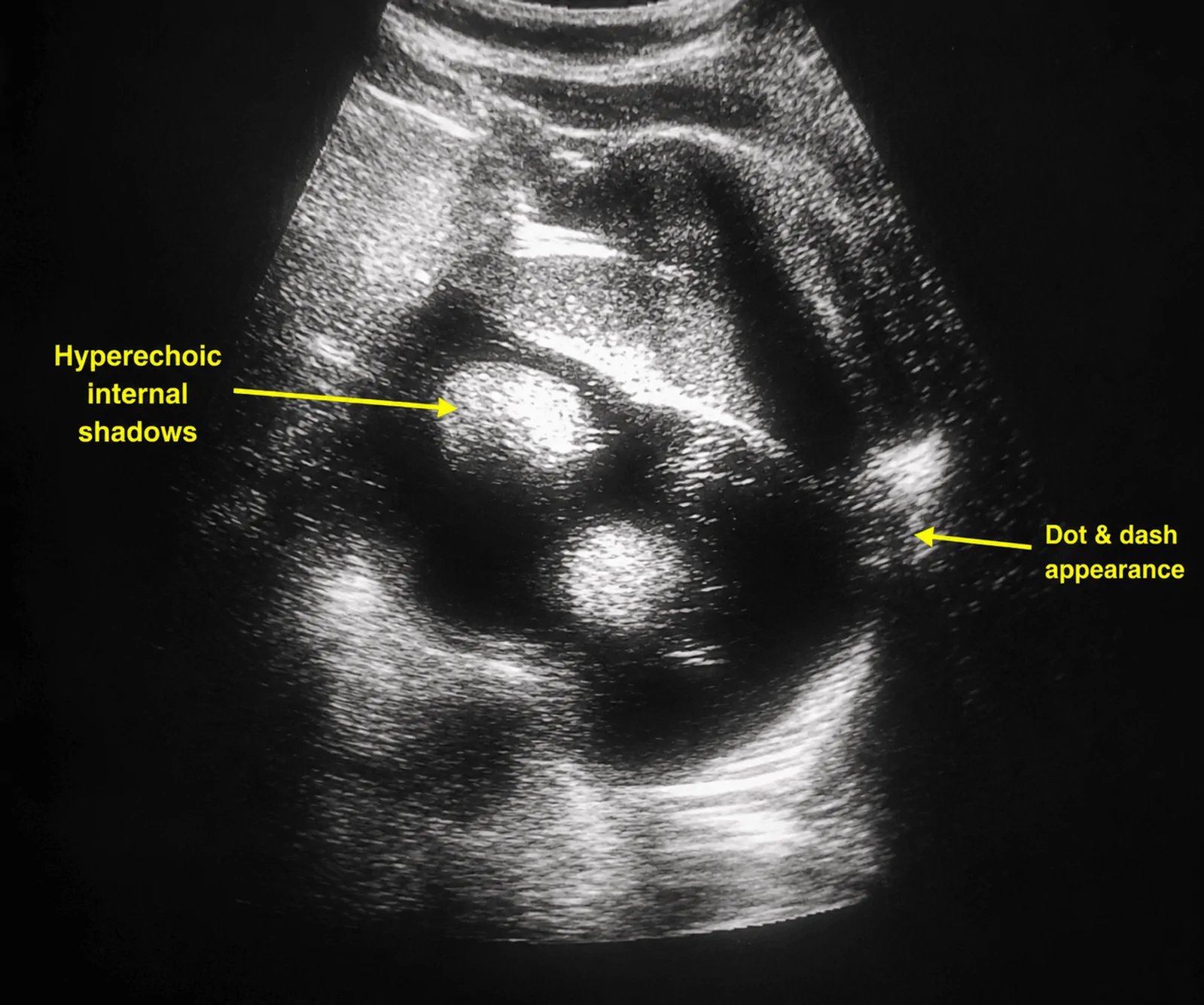
Transvaginal pelvic ultrasonography of the left adnexal mass. The figure demonstrates characteristic sonographic markers of a mature cystic teratoma (MCT), including floating hyperechoic internal foci representing sebaceous material/Rokitansky protuberance (left arrow) and the classic "dot-and-dash" pattern representing acoustic interfaces of hair shafts within thick fluid (right arrow).

In pelvic magnetic resonance imaging (MRI), a large and well-circumscribed mass of size 12.2 × 9.85 × 10.48 cm with fat-fluid levels and fat containing spherules was noted in the left ovary, indicating the presence of an MCT. There was no definite enhancing solid component. The mass led to indentation of the surrounding organs with preserved intervening fat planes. The right ovarian dermoid cyst with a smooth and thin enhancing periphery was also seen (Figure 2).

**Figure 2:**
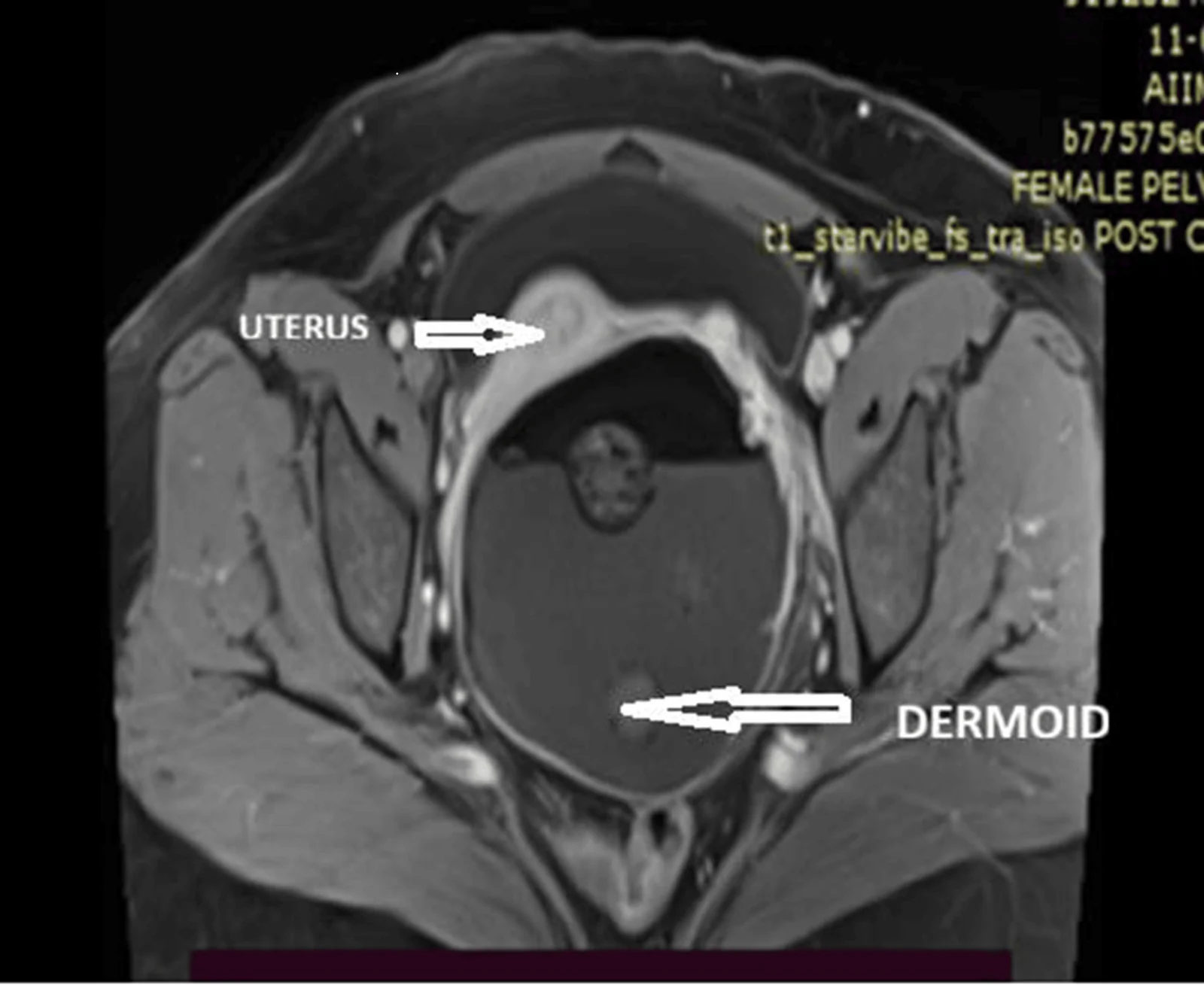
Axial T1-weighted contrast-enhanced pelvic MRI. The figure demonstrates a large, well-defined cystic pelvic lesion consistent with a dermoid cyst (mature cystic teratoma); the uterus is indicated by the arrow.

Most laboratory parameters were within the normal limits. The serum cancer antigen 125 (CA-125) level was elevated at 185.90 U/mL (reference range, 0-35 U/mL). The preoperative laboratory investigations are summarized in Table 1. 

**Table 1:** Preoperative laboratory investigations of the patient. Values are presented as measured laboratory values with the corresponding reference ranges. Reference ranges are based on the reference intervals provided by the institutional laboratory. Abbreviations: ALT, alanine aminotransferase; AST, aspartate aminotransferase; SGOT, serum glutamic-oxaloacetic transaminase; SGPT, serum glutamic-pyruvic transaminase; TSH, thyroid-stimulating hormone

Laboratory tests	Patient value	Reference range
Hemoglobin (g/dl)	11.8	12-15
Total leukocyte count (10˄3/mm³)	5.0	4.0-11.0
Platelet count (10˄6/mm³ )	1.9	1.5-4.5
Thyroid-stimulating hormone (mIU/L)	4.17	0.4-5.0
Urea (mg/dl)	20	13-40
Serum Creatinine (mg/dl)	0.6	0.6-1.1
Serum Albumin (g/dl)	4.2	3.5-5.2
Total Bilirubin (mg/dl)	0.8	0.3-1.0
SGOT (ALT) (U/ml)	18	< 34
SGPT (AST) (U/ml)	11	< 31
Pap smear	Negative for intraepithelial lesion or malignancy	--
Cancer antigen-125 (U/mL)	185.90	0-35

The patient was counselled and planned for laparotomy. Total abdominal hysterectomy with bilateral salpingo-oophorectomy (TAH-BSO) was performed.

Intraoperative findings included no ascites, a bulky uterus measuring approximately 8-10 weeks in size with myometrial hyperplastic changes. The endometrium and cervical canal were unremarkable. Both fallopian tubes appeared grossly normal. The right ovary was enlarged and contained an encapsulated, multiloculated cyst measuring 5 × 3 cm with a regular outer surface. The left ovary was enlarged and impacted in the pouch of Douglas and contained two cysts. The larger cyst was well encapsulated, measured 12 × 8 × 8 cm, and had a smooth surface, thinned-out cyst wall, and visible vascularity. The second cyst was well encapsulated, measured 2.5 × 3 cm, and had a smooth surface. The operated specimen is shown in Figure 3.

**Figure 3:**
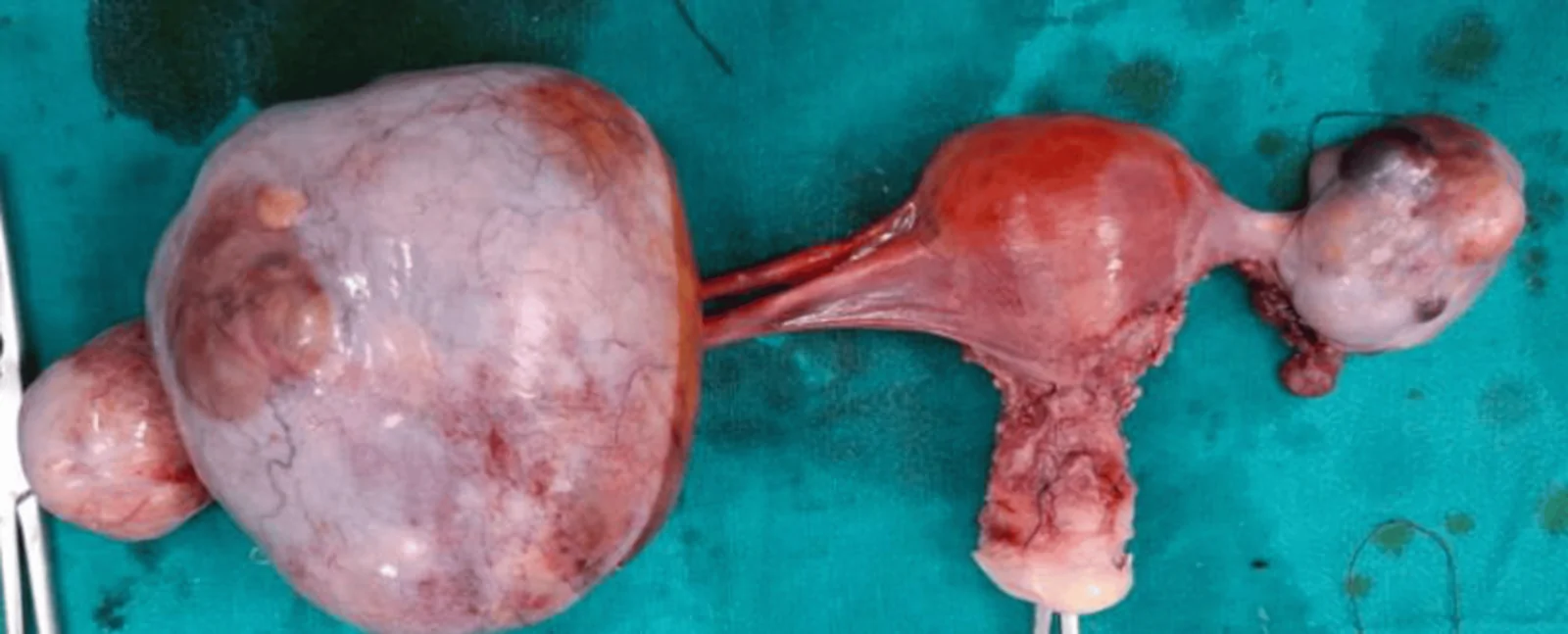
Gross pathology specimen following total abdominal hysterectomy and bilateral salpingo-oophorectomy. The central uterus and cervix are depicted alongside bilateral adnexa. The right/left adnexa demonstrates a prominently enlarged, smooth-surfaced, multilocular cyst fluid/mass lesion with prominent vascular markings on the capsule, consistent with an ovarian neoplasm (e.g., cystadenoma or mature cystic teratoma). The contralateral adnexa shows normal/mildly altered ovarian parenchyma.

The uterus exhibited hyperplastic changes in the myometrium on cut section. There was nothing significant to comment on in the endometrium and cervical canal. Both ovaries with the cystic masses were processed intact for histopathologic analysis. Histopathologically, there was an intact ovarian capsule with no ovarian capsule involvement. The left ovary had an MCT with malignant changes in the form of moderately differentiated SCC characterized by cohesive nests of pleomorphic squamous epithelial cells, prominent keratin pearl formation, and frequent mitotic figures. The right ovary had features of MCT with no malignant changes observed (Figure 4).

**Figure 4:**
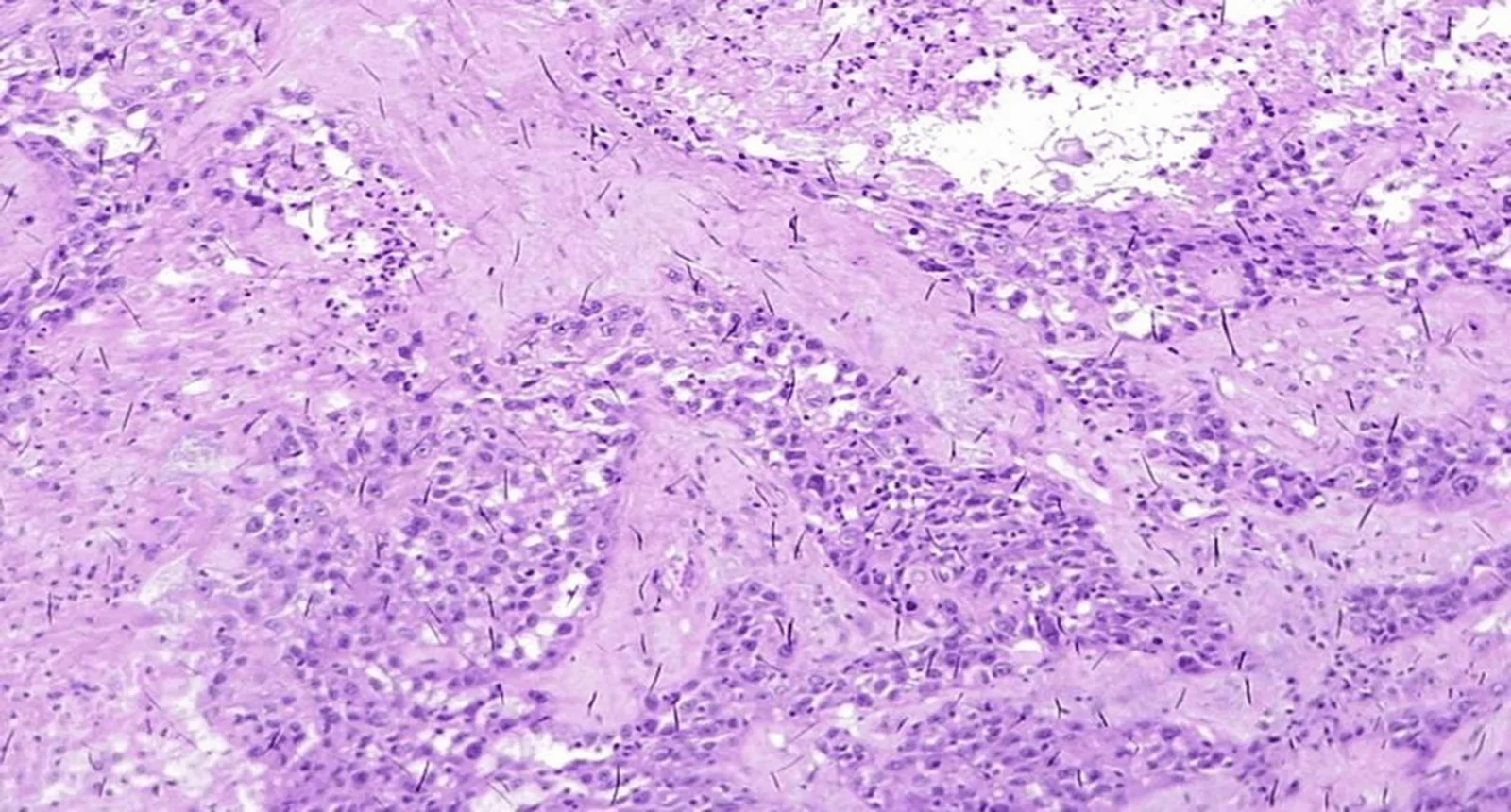
Histopathology of the left ovarian mass (H&E, ×40). Photomicrograph shows mature ectodermal elements of a cystic teratoma transitioning into islands of invasive, moderately differentiated squamous cell carcinoma (SCC) with cytological atypia and keratin pearl formation.

Tumor cells in the left ovarian lesion demonstrated strong, diffuse nuclear expression for both p63 and p40, confirming the squamous lineage (Figures 5, 6).

**Figure 5:**
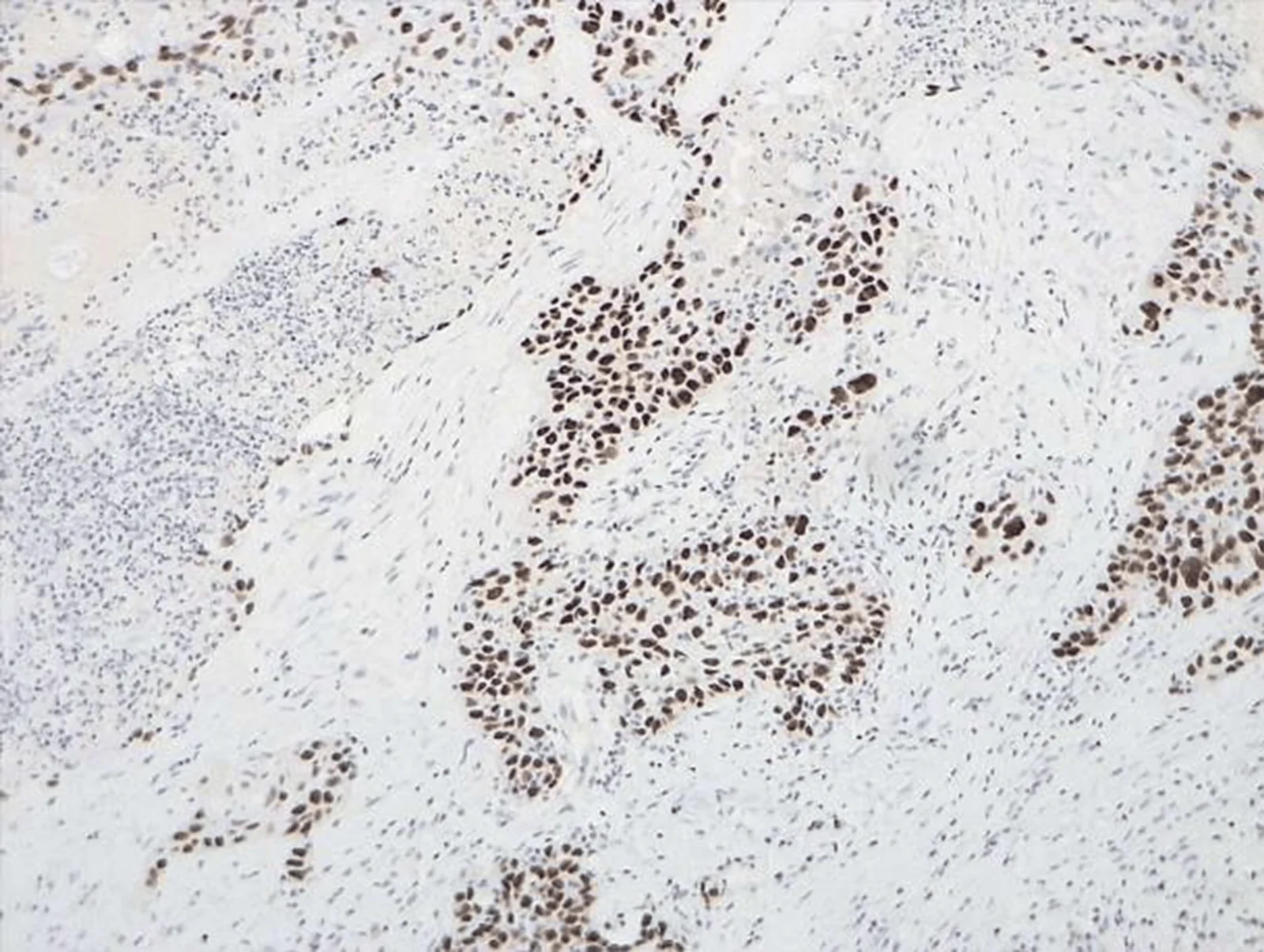
Immunohistochemical expression of p63 (×40). Photomicrograph demonstrating strong, diffuse nuclear immunoreactivity for p63 within neoplastic cell nests, confirming squamous lineage.

**Figure 6:**
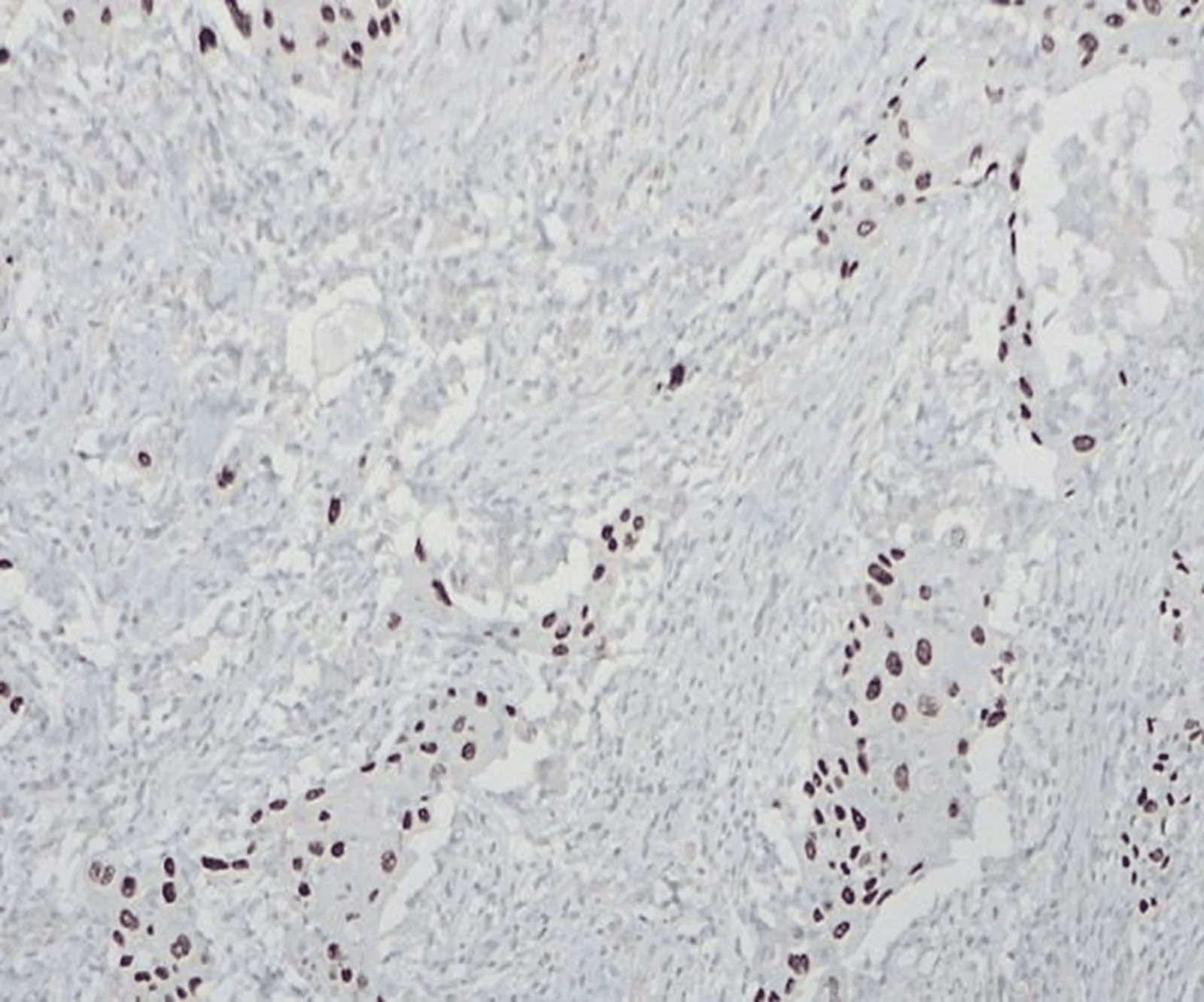
Immunohistochemical expression of p40 (×40). Photomicrograph showing high-intensity nuclear positivity for p40, confirming primary squamous cell carcinoma.

The postoperative recovery was uneventful. Following the histopathological diagnosis of malignant transformation to SCC, the patient was counselled regarding the potential need for formal completion surgical staging, including peritoneal washing cytology, omental biopsy, and pelvic and para-aortic lymphadenectomy. The patient declined secondary staging laparotomy.

A postoperative whole-body PET-CT scan demonstrated no FDG-avid nodal, peritoneal, or distant organ metastases. Serum CA-125 decreased to 35.0 U/mL. As formal surgical staging was not performed, a definitive FIGO pathological stage could not be assigned. Based on the available surgical, histopathological, and imaging findings, there was no evidence of extra-ovarian disease.

Following multidisciplinary tumour board consultation with gynecologic oncologists and radiation oncologists, adjuvant platinum-based combination chemotherapy was recommended in view of the malignant transformation to SCC and the absence of formal surgical staging. The patient was initiated on carboplatin (AUC 5) and paclitaxel (175 mg/m²), administered intravenously every three weeks. She is currently receiving her fourth cycle of chemotherapy and is tolerating treatment well without major adverse events. Serum CA-125 has subsequently normalized to 18.2 U/mL, and she remains under ongoing clinical follow-up without evidence of disease to date.

## Discussion

This case shows that it can be difficult to make a diagnosis when there is malignant transformation from an MCT of the ovary, especially since the imaging results still show a predominance of benign neoplasm.

There are certain clinical and imaging characteristics that have been linked to malignant potential in MCT, such as older age, post-menopausal condition, greater tumor size, solid components, and high tumor markers. In the present case, the following factors, such as the patient’s age of 46, her perimenopausal condition, a large left ovarian tumor of around 12 cm in diameter, and CA-125 of 185.9 U/mL, could be considered key clinical clues for potential malignancy [1,5,7]. The relation between age and tumor size has also been proven in clinicopathological investigations. According to a study conducted by Rathore et al. on 230 cases of MCT, there were eight cases (3.5%) of malignant transformation. The patients with malignant transformation were older than those with benign MCTs; the average age was 44.2 ± 8.94 and 32.5 ± 13.11 years, respectively. Moreover, the average size of the tumor was significantly larger in cases of malignant transformation (11.7 ± 2.7 cm) than in benign MCTs (7.6 ± 2.1 cm). Among different histological subtypes of MCT, SCC was the commonest one in cases of malignant transformation (four out of eight cases). These data strongly correspond with our patient with MCT (46 years of age, 12 cm tumor), which transformed to SCC [8].

Diagnosing malignant transformation preoperatively is difficult since the imaging results can be similar to benign MCT. In the current case, ultrasonography revealed typical features of bilateral dermoid cysts, such as the "dot and dash" sign, while MRI revealed fat-fluid level and fat-filled spheres characteristic of MCT. However, there were no signs of enhancement of the solid component. Even though large size of the tumor, irregular margins, wall thickening, and enhancing solid component have been mentioned as radiological signs that might suggest the presence of malignant transformation, the absence of these signs cannot entirely rule out the malignancy. In malignant transformation, it can be localized in one part of the tumor that is otherwise typical for MCT, and thus, it may be hard to diagnose preoperatively.

The histopathological analysis played an essential role in making a proper diagnosis. In the current case, the left ovarian tumor revealed a malignant transformation from an MCT to moderately differentiated squamous cell carcinoma, whereas the right ovarian lesion was a benign MCT. Positive expression for p63 and p40 indicated squamous differentiation and corresponded to histomorphological features. The malignant transformation of MCT may reveal different phenotypic types, based on the type of somatic tissue. Previously published literature revealed some rare cases of the co-existence of both epithelial and sarcomatous malignant components in teratomas. In the current case, only epithelial malignant transformation was observed in the form of moderately differentiated SCC. The presence of such a difference is of significance since SCC is the most frequent type of MCT malignant transformation, and sarcomatous transformation is much less frequent [9].

The molecular basis of malignant transformation within mature ovarian teratomas remains incompletely understood. Recent genomic studies have provided evidence supporting the germ cell origin of ovarian SCC arising from mature ovarian teratomas. In a molecular study of 11 ovarian SCCs arising from mature ovarian teratomas, molecular inversion probe array analysis demonstrated abnormalities consistent with failures of meiosis I, meiosis II, and endoreduplication, while these alterations were not observed in ovarian metastases of SCC originating from extraovarian primary sites. These findings support the concept that primary ovarian SCC arising within a teratoma may originate from teratoma-derived germ cells rather than representing metastatic SCC to the ovary. Targeted sequencing further identified TP53 as the most frequently mutated gene (82%), followed by PIK3CA (36%), PTEN (27%), and KMT2D (27%). Alterations involving CDKN2A and copy-number loss at 9p21.3 were also identified in a substantial proportion of cases. These findings provide important insights into the genomic landscape and biological origin of SCC arising within mature ovarian teratomas and may help distinguish primary ovarian SCC from metastatic squamous malignancy [10].

Prognosis in ovarian SCC is generally less favorable than that of other ovarian carcinomas and is strongly influenced by disease stage and clinicopathological characteristics. In a matched cohort study including 304 patients with ovarian carcinoma, older age at diagnosis, larger tumor size, bilateral disease, and FIGO stage IV disease were independently associated with poorer outcomes [11]. These findings are particularly relevant to the present case, as the patient was 46 years old and had a large 12-cm tumor with bilateral MCTs. However, the absence of metastatic disease on postoperative PET-CT is reassuring, and the prognostic implications should be interpreted cautiously because formal surgical staging was not performed.

Management of SCC arising from MCT remains challenging because of its rarity and the absence of prospective studies establishing a standardized treatment approach. Complete surgical excision with appropriate staging is generally considered the cornerstone of management [1]. In the present case, hysterectomy with salpingo-oophorectomy was done. But the patient refused to undergo any surgical staging after the diagnosis of SCC. The postoperative PET-CT revealed no metastasis, and the CA-125 reduced from 185.9 U/mL to 35 U/mL. Even though this is positive, a negative PET-CT is not a replacement for surgical staging and can never rule out any micrometastasis. Thus, the pathological stage of the FIGO system could not be defined in this patient.

This case brings out many significant clinical points. Malignant transformation needs to be kept in mind in a postmenopausal female with a large MCT, especially if she has elevated tumor markers. It must be noted that lack of definite enhancement of the solid component on imaging does not rule out malignant transformation. Histopathological assessment is very critical for the diagnosis, since it is possible for the malignant transformation to be focal in an otherwise normal dermoid cyst. It must be added that the presence of bilateral MCT, with one being malignant, also calls for a thorough evaluation of the ovaries. Lastly, since the evidence for SCC arising from MCT is limited, a treatment plan will have to be worked out by multidisciplinary discussion.

## Conclusions

Malignant transformation of MCT into SCC is a rare clinical entity. In perimenopausal women presenting with large adnexal masses (>10 cm), bilateral MCTs, elevated serum CA-125, and age >40 years, malignant transformation may warrant consideration. Histopathology and immunohistochemistry remain essential for establishing the diagnosis. Since complete surgical staging is not done in this case and there is a lack of long-term follow-up studies with respect to ovarian squamous cell carcinoma, interpretation of prognosis remains cautious, and continued long-term surveillance is warranted.
